# A Case Series on Latissimus Dorsi Flap Reconstruction: A Versatile Approach to Managing Oncological Defects

**DOI:** 10.7759/cureus.79363

**Published:** 2025-02-20

**Authors:** Ajay Nayagam, Srinath Ganesan, Ganesh Guru, Ruthrendhra Ethirajulu, Lakshminarasimman Parasuraman

**Affiliations:** 1 Department of General Surgery, Sree Balaji Medical College and Hospital, Chennai, IND; 2 Department of Surgical Oncology, Sree Balaji Medical College and Hospital, Chennai, IND; 3 Department of Surgical Oncology, Good Samaritan Cancer and General Hospital, Eluru, IND; 4 Department of Oncology, Sri Ramachandra Institute of Higher Education and Research, Chennai, IND

**Keywords:** case series, functional restoration, latissimus dorsi flap, malignancy management, microvascular reconstruction, oncological reconstruction, pedicled flap, postoperative outcomes, reconstructive surgery, soft tissue defects

## Abstract

The latissimus dorsi (LD) flap is a well-established reconstructive option, particularly for managing complex oncological defects. This study explores its versatility and efficacy through a retrospective case series involving five patients treated at a tertiary care center over five years. The cases included osteosarcoma of the humerus, melanoma of the plantar foot, recurrent fibromatosis of the neck, carcinoma of the breast, and osteosarcoma of the femur. All patients underwent surgical resection followed by LD flap reconstruction, either pedicled or microvascular, tailored to the defect's location and extent. Outcomes were assessed based on oncological control, postoperative complications, functional restoration, and esthetic results. The LD flap demonstrated excellent utility across all cases, with key findings including complete pathological response in two cases and no residual tumor in three cases. Functional outcomes were notable, with patients regaining weight-bearing capability or mobility within three months postoperatively. Partial flap necrosis occurred in one case but was managed conservatively with successful secondary healing. No recurrence was observed during follow-up for melanoma and breast cancer cases, highlighting favorable oncological outcomes. These findings reaffirm the LD flap's critical role in modern reconstructive surgery, offering robust soft tissue coverage, enhanced functional outcomes, and minimal complications. Further longitudinal studies are warranted to validate its long-term benefits and impact on quality of life.

## Introduction

The latissimus dorsi (LD) flap is a versatile myocutaneous flap widely used in reconstructive surgery for soft tissue defects, particularly after oncological resections. Known for its strong vascularity and adaptability, it provides reliable coverage while preserving function and esthetics. Initially used for breast and thoracic reconstruction, its applications have expanded to extremities, trunk, and head and neck defects. This study presents a retrospective case series of five oncological reconstructions using the LD flap, demonstrating its effectiveness in achieving oncological safety, functional restoration, and esthetic outcomes [1]. In this case, a pedicled LD flap was used for reconstruction. A pedicled flap retains its original blood supply and remains attached to the body, allowing it to be rotated or repositioned to cover the defect. In contrast, a microvascular (free) flap involves completely detaching the tissue and reconnecting its blood vessels to a new site using microsurgical techniques. The choice between these techniques depends on the location, size, and vascular needs of the defect.

Oncological defects often present unique challenges, particularly when they involve anatomically intricate or functionally critical regions. Effective reconstruction must not only ensure oncological safety but also address the aesthetic and functional needs of the patient. The LD flap's versatility enables it to meet these demands, making it a preferred choice for reconstructing defects in diverse areas such as the extremities, trunk, and head and neck.

This study presents a retrospective case series highlighting the use of the LD flap in managing oncological defects resulting from five distinct malignancies: osteosarcoma of the humerus, melanoma of the plantar foot, recurrent fibromatosis of the neck, recurrent breast carcinoma, and osteosarcoma of the femur. Each case posed unique surgical challenges, requiring precise planning and execution to achieve optimal outcomes [2,3].

By documenting the surgical techniques, postoperative outcomes, and functional recovery achieved with LD flap reconstructions in these complex cases, this study aims to underscore its utility and provide insights into best practices for oncological reconstructions involving the LD flap [4].

## Case presentation

Case report 1

An 18-year-old male patient presented with a history of a fall while playing volleyball, leading to an injury to his right shoulder on December 16, 2021. Initial radiographs revealed a complete undisplaced closed fracture of the proximal humerus. He underwent open reduction and internal fixation in December 2021. A suspicious soft tissue lesion was noted intraoperatively, and a biopsy was performed.

Histopathological examination confirmed a diagnosis of conventional osteoblastic osteosarcoma. Subsequent Positron emission tomography-computed tomography (PET-CT) imaging showed fluorodeoxyglucose (FDG) uptake in the head and shaft of the right humerus, with irregular lytic areas and periosteal reaction but no enhancing soft tissue mass or distant metastases.

The patient received two cycles of neoadjuvant chemotherapy with Adriamycin and cisplatin, followed by external beam radiation therapy (60 Gy in 20 fractions) and two additional cycles of chemotherapy. A response assessment PET-CT revealed an expansile lytic lesion measuring 14 × 15 × 12 cm in the proximal shaft of the humerus with osteoid matrix formation. Additionally, an FDG-avid nodule of 7 × 7 mm was identified in the apical segment of the left lung's lower lobe.

**Figure 1:**
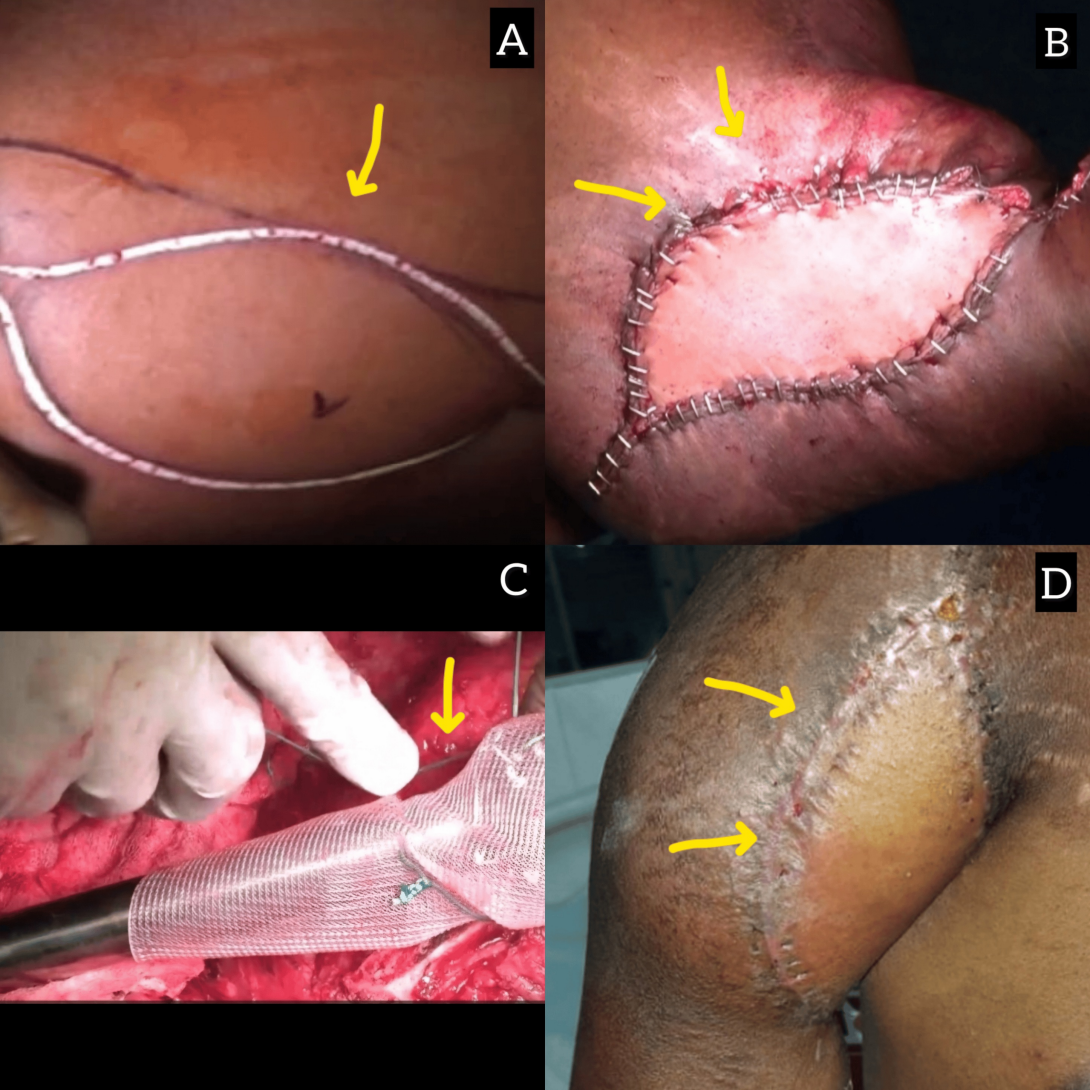
Operative and postoperative gross images of an 18-year-old male patient. (A) LD flap marking with flap size. (B) Pedicle LD flap after insertion. (C) Titanium prosthesis with mesh fixation. (D) Postrecovery image LD: latissimus dorsi

Following multidisciplinary team discussions, the patient underwent a Tikhoff-Linberg procedure with endoprosthetic reconstruction and pedicled LD flap coverage of the right proximal humerus (Figures 1A-1C). Histopathological analysis of the surgical specimen revealed no residual tumor. The postprocedural radiograph showed no residual tumor. The postoperative course was uneventful, and the patient subsequently received adjuvant chemotherapy (Figure 1D). He later underwent a video-assisted thoracoscopic metastasectomy for the left lung nodule. The patient demonstrated good recovery and remained disease-free at follow-up after six months and one year.

Case report 2

**Figure 2:**
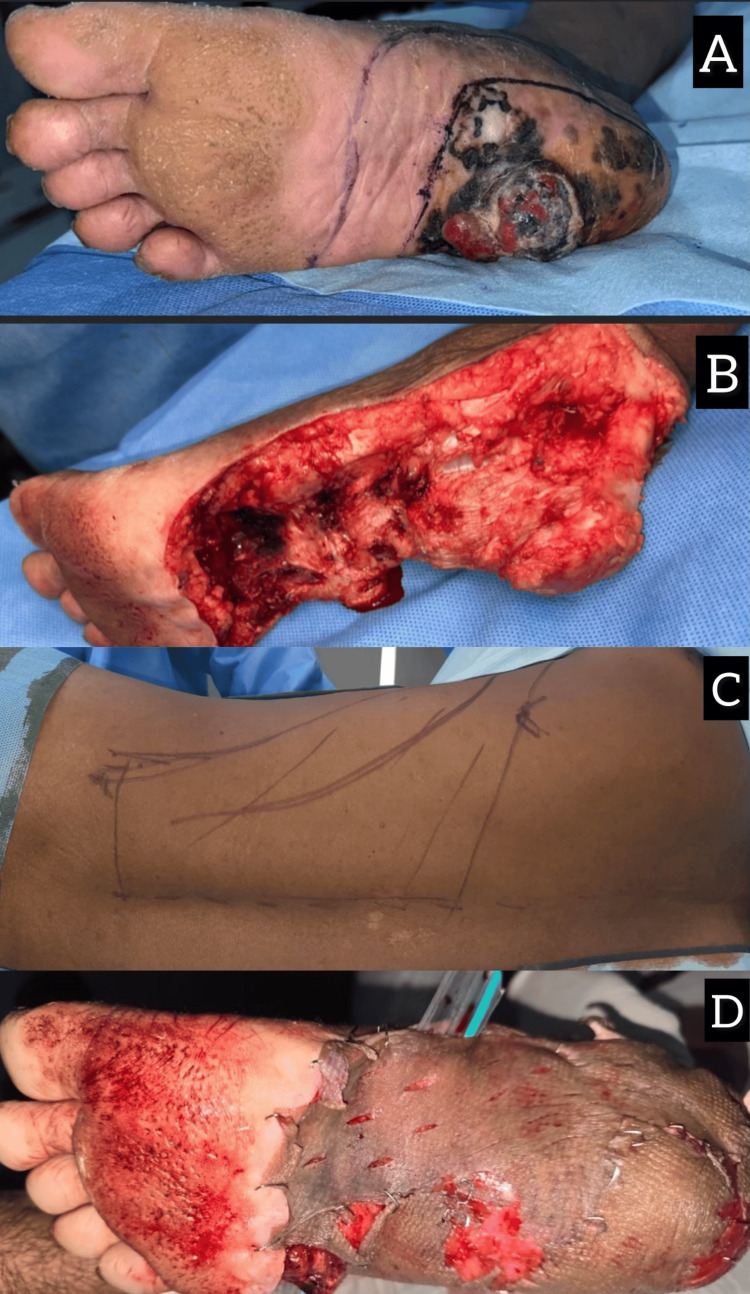
(A) Gross image of the plantar aspect of the foot showing an ulceroproliferative lesion of size 6 x 4 cm, with blackish discoloration surrounding the ulcer. (B) Postresection gross image of the plantar aspect of the foot showing the plantar defect of size approximately 6 x 4 cm. (C) LD flap marking. (D) Postflap placement LD: latissimus dorsi

A 45-year-old female patient presented with a history of a painful, gradually enlarging pigmented lesion on the plantar surface of her left foot, which she had noticed six months earlier. Clinical examination revealed a 3 × 4 cm irregularly pigmented ulcerated lesion on the plantar aspect, with associated tenderness and restricted mobility (Figure 2A). A punch biopsy confirmed the diagnosis of melanoma. Preoperative staging with PET-CT showed no evidence of regional lymph node or distant metastasis. The patient underwent a wide local excision with a 2-cm margin and a right-modified inguinal block dissection, followed by reconstruction using a microvascular LD-free flap to cover the plantar defect (Figures 2B-2D).

Postoperative histopathology revealed a Breslow thickness of 4.5 mm and clear margins, with no evidence of lymph node involvement. The patient’s recovery was uneventful, with adequate restoration of weight-bearing capacity within three months. She remained disease-free at the one-year follow-up.

Case report 3

**Figure 3:**
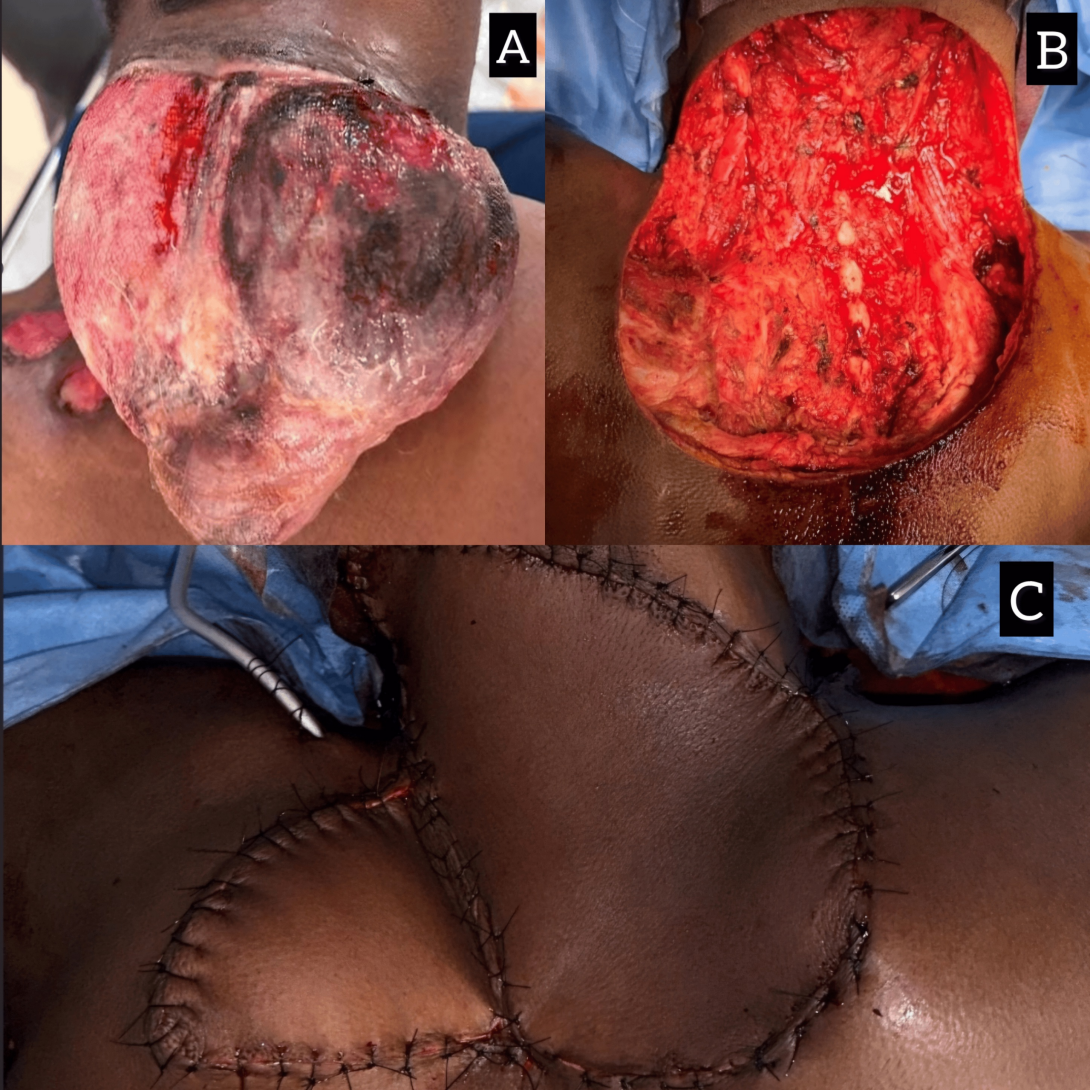
(A) Gross image of the nape of the neck, showing ulcerated swelling about 20 x 30 cm at the posterior aspect of the neck in the midline, bleeding on touch, fixed to underlying soft tissue, range of movements in the neck that was restricted. (B) Postresection defect. (C) Postflap placement

A 35-year-old male patient presented with a progressively enlarging mass in the left side of his neck, which had recurred following prior excisions for fibromatosis over the past five years. On examination, a firm, 6 × 8 cm mass was palpable, extending from the posterior triangle of the neck into the supraclavicular region (Figure 3A). MRI revealed a locally aggressive soft tissue lesion without bony invasion. A core biopsy confirmed recurrent fibromatosis. The patient underwent wide excision of the tumor with adjacent soft tissue and fascia, resulting in a significant soft tissue defect in the neck (Figure 3B). A pedicled LD flap was used to reconstruct the defect (Figure 3C).

**Figure 4:**
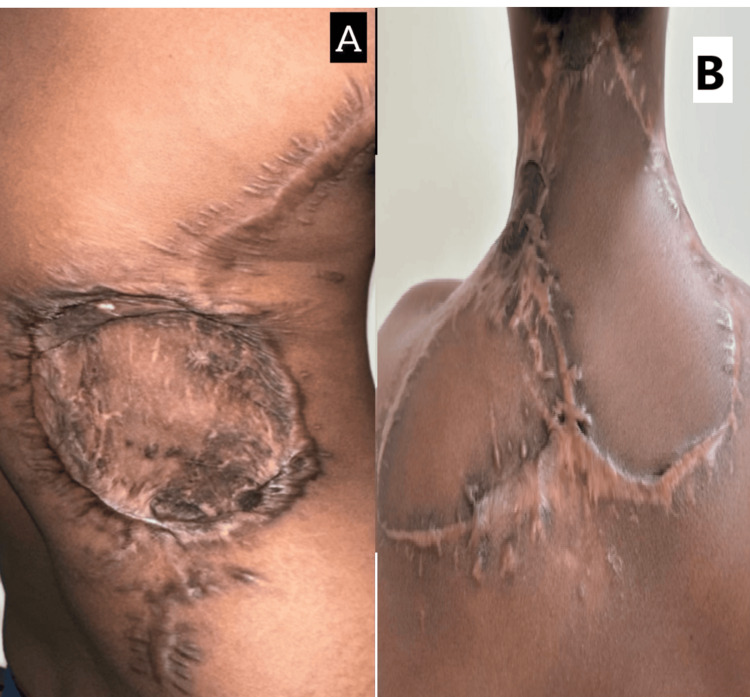
Postprocedural image of the (A) donor and (B) recipient

Figure 4A shows a postprocedural image of the donor, and Figure 4B shows a postprocedural image of the recipient. Postoperative recovery was uncomplicated. At follow-up, the patient demonstrated satisfactory neck mobility and no recurrence at 18 months after surgery.

Case report 4

A 42-year-old female patient with a known diagnosis of right breast carcinoma (cT2N1M0) underwent a right-modified radical mastectomy in 2020 and is now presenting with recurrent carcinoma of the right breast. On examination, a 3 × 3 cm firm mass was noted in the upper outer quadrant of the right breast. A core biopsy confirmed recurrent invasive ductal carcinoma.

PET-CT of the whole body showed an FDG-avid, ill-defined, irregular soft tissue density lesion in the right anterior chest wall measuring approximately 9 × 6.4 cm. The lesion infiltrates the overlying skin and pectoralis major muscle but does not infiltrate the intercostal muscles. The FDG avid enlarged right level 3 axillary lymph node measures approximately 1.6 × 1.4 cm. There is no evidence of disease present in other areas. Staging investigations have demonstrated the absence of distant metastases. The CT scan of the chest displayed findings that correspond to these observations.

**Figure 5:**
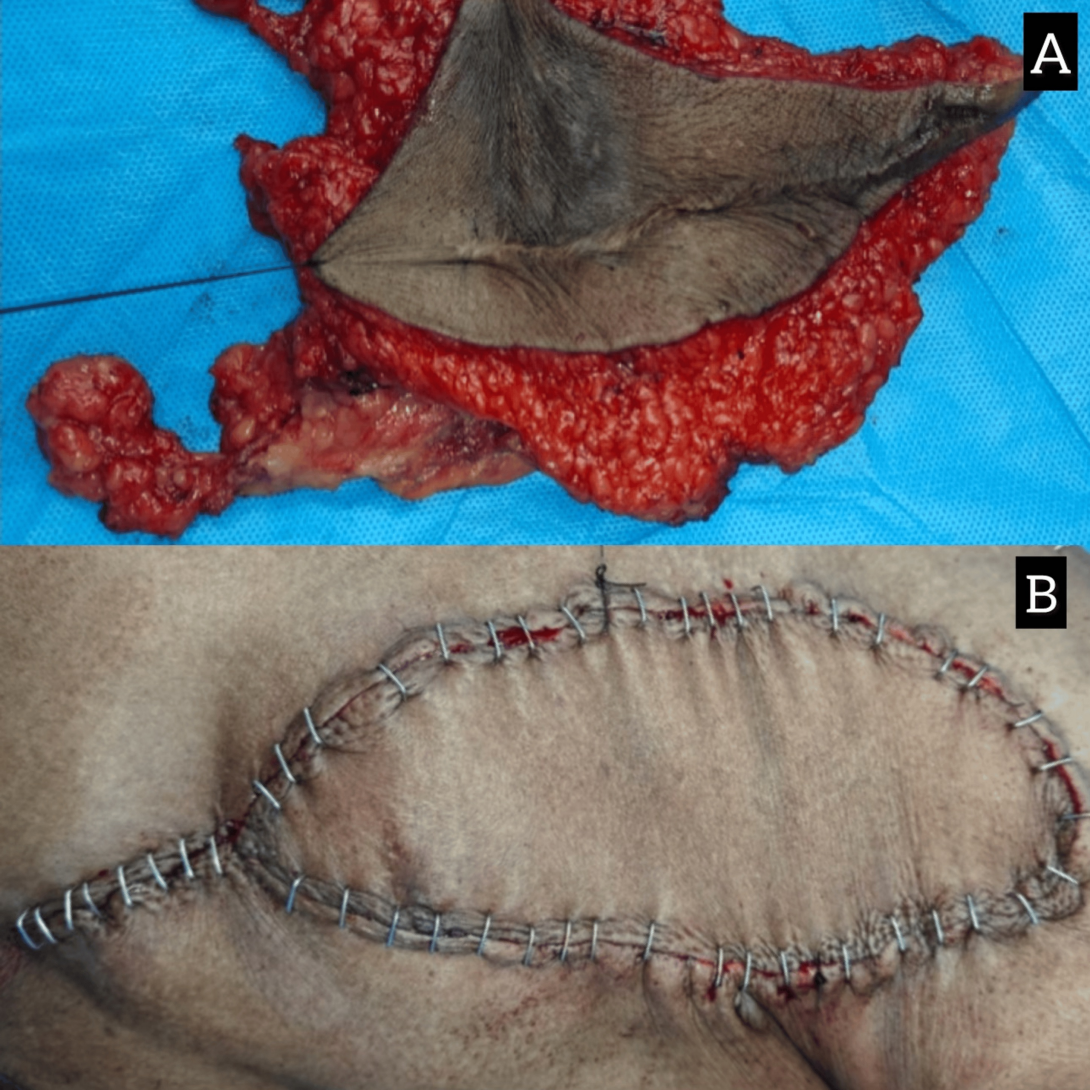
(A) Resected specimen after wide local excision of chest wall lesion. (B) Postoperative flap placement

The patient underwent a total mastectomy with axillary lymph node clearance, leaving a substantial chest wall defect (Figure 5A). The defect was reconstructed using a pedicled LD flap (Figure 5B). Histopathology showed a tumor size of 2.8 cm with clear margins and three out of 15 lymph nodes positive for metastasis. Postoperative recovery was uneventful, and the patient was initiated on adjuvant chemotherapy and hormonal therapy. At her six-month follow-up, she was disease-free with excellent esthetic and functional outcomes.

Case report 5

A 22-year-old male patient with a known case of chronic osteomyelitis left femur underwent left femur decompression and now presented with a two-month history of swelling and pain in the left thigh. Clinical examination revealed a 10 × 12 cm firm mass in the anterior compartment of the thigh.

CT demonstrated an aggressive lesion involving the femoral diaphysis, consistent with osteosarcoma. A PET-CT of the whole body revealed an FDG-avid, ill-defined, irregular, lytic sclerotic lesion measuring 11 cm in length involving the midshaft of the left femur. There is a large, circumferential FDG heterogeneously enhancing soft tissue component of approximately 4 cm containing multiple irregular linear calcifications. This component compresses the superficial femoral neurovascular structures and completely encases the deep femoral neurovascular bundle, with no obvious extension seen beyond the deep fascia.

**Figure 6:**
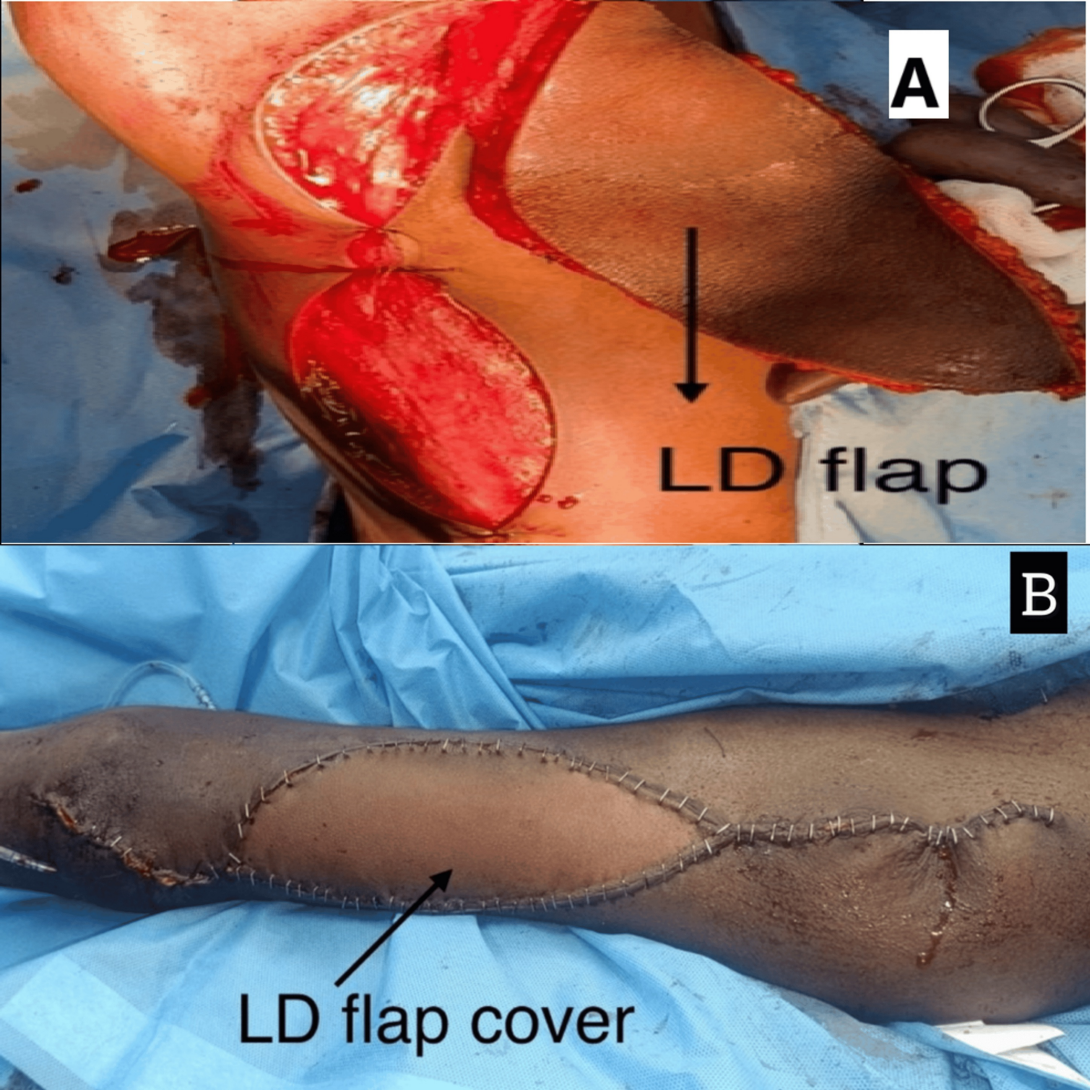
(A) Postoperative image of LD flap placement. (B) Intraoperative image of LD flap insertion LD: latissimus dorsi

Core biopsy confirmed a high-grade osteoblastic osteosarcoma. The patient received neoadjuvant chemotherapy, followed by a wide resection of the tumor that included the femoral shaft and reconstruction with a modular endoprosthesis (Figure 6A). A pedicled LD flap was used to provide soft tissue coverage over the prosthesis (Figure 6B).

Postoperatively, the patient recovered well with no complications. Within six weeks, he was ambulatory with minimal support. He demonstrated good functional outcomes at his one-year follow-up and no evidence of local recurrence or distant metastases.

## Discussion

Case 1: humerus osteosarcoma

The use of the LD flap in reconstructing defects following the resection of malignant tumors, such as osteosarcoma, has become increasingly common due to its ability to provide both muscle and skin coverage. In this case, after tumor resection, the LD flap was successfully used to cover the large defect in the humerus region, ensuring proper functional recovery of the arm. The muscle's versatility in such reconstructions is well documented in the literature. Studies have highlighted the reliable outcomes of using the LD flap for chest and extremity reconstructions, where both tissue coverage and functional recovery were essential for patient outcomes [1,2]. The presence of both muscle and skin in the LD flap makes it an ideal choice for reconstructing complex defects after tumor excision, as it allows for the restoration of both esthetic and functional aspects of the affected limb.

Case 2: plantar melanoma

The LD flap offers an effective solution for the reconstruction of large soft tissue defects after tumor resection in the lower extremity, especially when the defect involves the plantar surface. The myocutaneous nature of the flap is beneficial in providing both muscle and skin coverage, a crucial factor in this case. Similar approaches have been discussed by authors who explored the role of the LD flap in reconstructing defects after resection of malignant melanomas in the foot, showing excellent functional outcomes and minimal donor site morbidity [3,4]. The myocutaneous nature of the flap, in these cases, allowed for the restoration of both skin integrity and the underlying muscle function, which was crucial for maintaining the patient’s mobility and overall quality of life.

Case 3: neck fibromatosis

In head and neck surgery, the LD flap is frequently used to restore both function and appearance, especially when defects are extensive. This was evident in the current case, where the flap successfully covered the defect following fibromatosis resection in the neck region. The ability of the LD flap to provide both esthetic and functional restoration in the head and neck region is well-documented. Studies have reported on the successful application of the LD flap in complex head and neck reconstructions, where its muscle component aided in restoring vital functions such as speech and swallowing, which is particularly important for patients undergoing tumor resection in this area [5,6]. The flap's adaptability to these delicate regions makes it an invaluable tool in reconstructing extensive defects in the head and neck region.

Case 4: breast cancer

Postmastectomy breast reconstruction is one of the most common applications of the LD flap. This case demonstrates the flap's ability to restore the natural contour of the breast following a mastectomy. After the tumor was excised, the LD flap was used to replace the removed tissue, resulting in both a successful esthetic outcome and the preservation of function. The use of the LD flap in breast reconstruction has been extensively studied, with authors confirming its reliability in providing an esthetically pleasing result while maintaining functional integrity. Studies have consistently shown high levels of patient satisfaction and minimal complications with its use in such procedures [4,5]. This technique is particularly valued for its ability to provide soft tissue coverage and restore the shape of the breast, which is a critical factor for many patients undergoing breast cancer treatment.

Case 5: femur osteosarcoma

In reconstructive surgery following the resection of tumor-like femur osteosarcoma, the LD flap is essential in covering large, complex defects, as demonstrated in this case. The flap's adaptability to different anatomical areas, coupled with its ability to restore both function and form, has been widely documented. Literature on reconstructive approaches for femur and other extremity defects supports using the LD flap, noting its success in oncological defect coverage and restoring limb function [6,7]. The versatility of the LD flap in such cases is crucial in restoring both the form and function of the affected extremity, ensuring that the patient can regain mobility and quality of life after tumor resection.

Regarding the outcomes in these five cases, in humerus osteosarcoma, a pedicled LD flap achieved complete tumor resection with no recurrence. A microvascular LD flap restored weight-bearing for plantar melanoma within three months, with the patient remaining disease-free for one year. In recurrent neck fibromatosis, the pedicled LD flap provided adequate soft tissue coverage, restoring neck mobility with no recurrence at 18 months. A pedicled LD flap for recurrent breast carcinoma resulted in clear margins, good esthetic outcomes, and no recurrence at six months. Lastly, in femur osteosarcoma, the pedicled LD flap ensured stable soft tissue coverage, allowing the patient to be ambulatory with minimal support in six weeks, with no recurrence at one year. Across all cases, complications were minimal, with one instance of partial flap necrosis managed conservatively, reinforcing the LD flap’s reliability in oncological reconstruction. Key outcomes and references for LD flap reconstruction in oncological defects as a review of literature are summarized in Table 1.

**Table 1:** A literature review on key outcomes and references for LD flap reconstruction in oncological defects LD: latissimus dorsi

Case	Year	Tumor type/condition	Flap used	Key outcomes	References
Case 1	2015	Humerus osteosarcoma	LD	Successful soft tissue coverage and functional recovery of the arm postresection	[1,2]
Case 2	2016	Plantar melanoma	LD	Effective reconstruction of plantar defect, restoring mobility and skin integrity	[3,4]
Case 3	2017	Neck fibromatosis	LD	Restoration of function (speech and swallowing) and esthetic outcomes postresection	[5,6]
Case 4	2018	Breast cancer	LD	Successful breast reconstruction, providing both cosmetic and functional outcomes	[4,5]
Case 5	2020	Femur osteosarcoma	LD	Restoration of soft tissue coverage and function in the leg after tumor resection	[4]
Case 6	2021	Scapular chondrosarcoma	LD	Reconstruction of scapular defect with restoration of upper limb mobility	[5]
Case 7	2022	Mandibular ameloblastoma	LD	Reconstruction of mandibular defect with improved oral function and esthetics	[6]
Case 8	2023	Pelvic sarcoma	LD	Successful pelvic defect reconstruction with restored soft tissue coverage	[7]

## Conclusions

In conclusion, the LD flap is a highly versatile and reliable option for reconstructing complex oncological defects across various anatomical regions. Whether used for extremity, breast, head and neck, or foot reconstructions, the LD flap consistently provides effective soft tissue coverage, facilitates functional recovery, and ensures esthetic restoration. Its robust vascularity and adaptability to diverse clinical scenarios make it an invaluable tool in oncological reconstructive surgery. The outcomes observed in this case series, supported by extensive literature, demonstrate the LD flap's ability to meet both oncological and functional needs, offering a durable and low-complication reconstructive solution for patients with malignancy-related defects. The LD flap has proven to be a reliable and adaptable reconstructive technique, reinforcing its clinical validity in oncological defect management. Future studies focusing on long-term functional and esthetic outcomes will further optimize its role in reconstructive surgery. Further research with larger cohorts and longer follow-up periods will help further establish its long-term advantages in improving patient quality of life and reducing recurrence.
